# Extracellular Prolidase (PEPD) Induces Anabolic Processes through EGFR, β_1_-integrin, and IGF-1R Signaling Pathways in an Experimental Model of Wounded Fibroblasts

**DOI:** 10.3390/ijms22020942

**Published:** 2021-01-19

**Authors:** Weronika Baszanowska, Magdalena Misiura, Ilona Oscilowska, Jerzy Palka, Wojciech Miltyk

**Affiliations:** 1 Department of Medicinal Chemistry, Medical University of Bialystok, Kilińskiego 1, 15-089 Bialystok, Poland; w.baszanowska22@wp.pl (W.B.); ilona.zareba@gmail.com (I.O.); pal@umb.edu.pl (J.P.); 2 Department of Analysis and Bioanalysis of Medicines, Medical University of Bialystok, Kilińskiego 1, 15-089 Bialystok, Poland; magdalena.misiura@umb.edu.pl

**Keywords:** β_1_-integrin, EGFR, fibroblasts, IGF-1, PEPD, prolidase, wound healing

## Abstract

The role of prolidase (PEPD) as a ligand of the epidermal growth factor receptor (EGFR) was studied in an experimental model of wound healing in cultured fibroblasts. The cells were treated with PEPD (1–100 nM) and analysis of cell viability, proliferation, migration, collagen biosynthesis, PEPD activity, and the expressions of EGFR, insulin-like growth factor 1 (IGF-1), and β_1_-integrin receptor including downstream signaling proteins were performed. It has been found that PEPD stimulated proliferation and migration of fibroblasts via activation of the EGFR-downstream PI3K/Akt/mTOR signaling pathway. Simultaneously, PEPD stimulated the expression of β_1_-integrin and IGF-1 receptors and proteins downstream to these receptors such as FAK, Grb2, and ERK1/2. Collagen biosynthesis was increased in control and “wounded” fibroblasts under PEPD treatment. The data suggest that PEPD-induced EGFR signaling may serve as a new attempt to therapy wound healing.

## 1. Introduction

Skin, as the largest organ of the human body, is responsible for several major functions. It protects against mechanical damage, extreme temperature, microbial infection, radiation, and other environmental factors [1]. Damage of this protective barrier may lead to serious disturbances in the functioning of the entire body. For this reason, the repair processes of this organ are of particular research interest. Skin repair requires the participation of several different cell types [2] as well as many different factors, cellular proteins, soluble growth factors, and extracellular matrix (ECM) proteins [3]. In this process fibroblasts and growth factors play a dominant role. It is well established that during the wound healing process fibroblast functions are activated by PDGF, EGF, IGF-1, and others [4]. However, not all defects in wound healing could be explained based on the growth factors. An example is prolidase deficiency (PD) that is accompanied, among others, by ulceration and impaired wound healing [5]. It got attention on the role of prolidase in wound healing, particularly because of recent data showing that prolidase operates as a regulator of epidermal growth factor receptor (EGFR) and HER2-dependent signaling pathways [6], p53 transcriptional activity [7], and interferon α/β receptor expression [8,9]. 

Prolidase, known as peptidase D (PEPD), plays a significant role in collagen metabolism and matrix remodeling. It participates in the last step of collagen degradation, cleaving dipeptides with proline or hydroxyproline at the C terminus. Released proline could be used for collagen resynthesis [10,11,12,13]. It has been discovered that PEPD is involved in the regulation of collagen biosynthesis. Using cellular models it has been shown that collagen-prolidase axis is affected in fibroblast treated with anti-inflammatory drugs [14], pyrroline 5-carboxylate (proline metabolite) [15,16], in experimental inflammation of chondrocytes [10], during experimental fibroblasts aging [15], activation of integrin receptor for type I collagen in osteogenesis imperfecta-derived fibroblasts [17] and cancer cell models [18,19,20].

EGFR is a member of the transmembrane receptor tyrosine kinases (ERBB family) which requires dimerization of receptor subunits for autophosphorylation inducing further signaling [21,22]. This process leads to the induction of proliferation, differentiation, and migration of the cells through the phosphorylation of proteins in several signaling pathways [23,24,25]. The key EGFR downstream signaling involves the PI3K/AKT/mTOR, Ras/Raf/ERK, and JAK/STAT pathways [6,26,27]. EGFR signaling is coupled to adhesion receptor signaling. Growth promoting and anabolic pathways require activation of integrin receptor pathways. For instance, stimulation of β_1_-integrin receptor induces autophosphorylation of FAK that integrates the signal from growth factor receptors leading to up-regulation of two MAP kinases: ERK1/2 [28,29] inducing cell growth, differentiation, and metabolism [28,30]. Of special interest is that activation of β_1_-integrin receptor up-regulates PEPD activity and collagen biosynthesis [8,9,12,31]. However, the role of PEPD in the anabolic and growth-promoting processes in tissue regeneration is still unknown.

We hypothesized that PEPD-EGFR interaction may represent an important mechanism for regenerative processes in the skin. Skin fibroblasts characterized by high PEPD activity and the capacity to synthesize collagen [8] served as model cells to study the effect of PEPD on several processes accompanied by experimental wound healing.

## 2. Results

### 2.1. The Viability of Fibroblasts Is Not Affected by Extracellular PEPD

**Figure 1:**
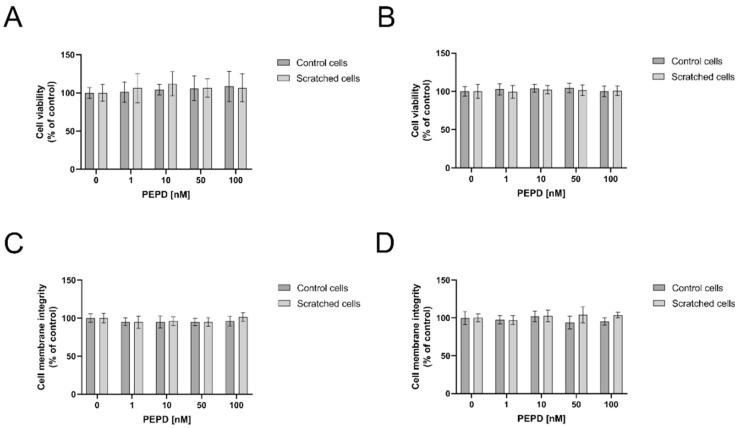
The effect of PEPD on fibroblasts viability and cell membrane integrity. Control cells, as well as scratched fibroblasts, were treated with PEPD (1–100 nM) for 24 h (**A**,**C**) and 48 h (**B**,**D**) followed by measurement of MTT and cell membrane integrity, respectively. Mean values ± SD of three experiments done in replicates are presented. The results are significant at a, b < 0.05 indicates a vs. control (0 nM of PEPD) of control cells, b vs. control (0 nM of PEPD) of scratched cells, respectively. PEPD—prolidase.

The effect of PEPD on cell viability was evaluated by measurement of MTT (methyl thiazolyl tetrazolium) concentration (Figure 1A,B) and the integrity of the cell membrane (Figure 1C,D). PEPD-treated fibroblasts (range of concentration: 1–100 nM) did not affect cell viability as well as cell membrane integrity in both cell models, control and “scratched”, after 24 and 48 h of incubation, respectively. 

### 2.2. Fibroblast Proliferation and Migration Are Augmented by PEPD Treatment in an Experimental Model of Wounded Cells

**Figure 2:**
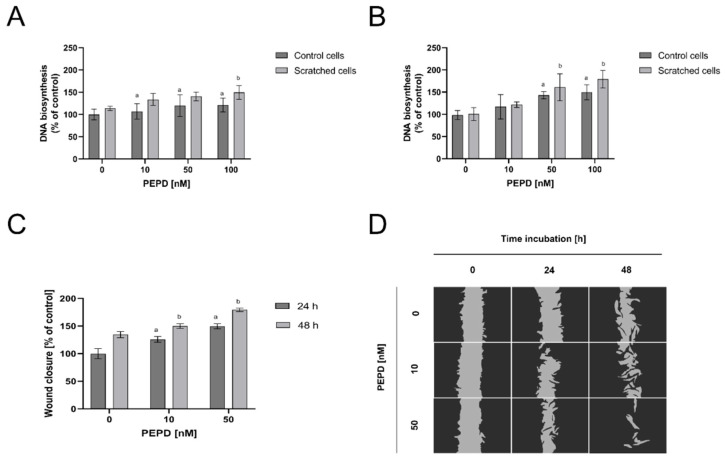
Extracellular PEPD-dependent proliferation and migration of fibroblasts in a model of closure/scratch assay. (**A**,**B**) Control, as well as “scratched” fibroblasts, were treated with PEPD (1–100 nM) for 24 h and 48 h, and proliferation was evaluated using CyQuant Proliferation assay. (**C**,**D**) PEPD-stimulated fibroblasts migration was calculated using ImageJ software (https://imagej.nih.gov/ij/) vs. control. PEPD-treated cells were scratched and monitored using an inverted microscope (40× magnification) at 0, 24, and 48 h. Mean values ± SD of three experiments done in replicates are presented. The results are significant at a, b < 0.05, and indicates a vs. control (0 nM of PEPD) of control cells and part C of 24 h incubation, b vs. control (0 nM of PEPD) of scratched cells, and part C of 48 h incubation, respectively. PEPD—prolidase.

The effect of PEPD on fibroblast proliferation after mechanical damage, the of the cell monolayer (by scratch), and DNA biosynthesis were measured by fluorescence assay. The cells were incubated with the selected concentrations (from 1 nM to 100 nM) of PEPD for 24 and 48 h. As shown in Figure 2A, PEPD promoted similar cell proliferation in both controls and “wounded” cellular models. The process was also time-dependent since after 48 h incubation (Figure 2B) the effect was augmented in both models of the cells. 

Similarly, the effect of extracellular PEPD on fibroblast migration was studied in wound closure/scratch assay. The data demonstrate that PEPD induced wound closure and the process was dependent on the dose of PEPD and time of incubation (Figure 2C). Quantification of the wounded area showed that PEPD-treated fibroblasts migrated faster to the wounded area than control cells, especially after 48 h incubation. These results were confirmed by microscopic visualization, as presented in Figure 2D. PEPD-treated cells for 48 h yielded nearly the entire closure of the wound, whilst the scratched area of control cells (no PEPD treatment) was slightly decreased. The obtained results showed that PEPD-driven wound closure is dependent on a dose and time of fibroblasts treatment with the EGFR ligand.

### 2.3. Extracellular PEPD Induces EGFR-Downstream Signaling Pathways

**Figure 3:**
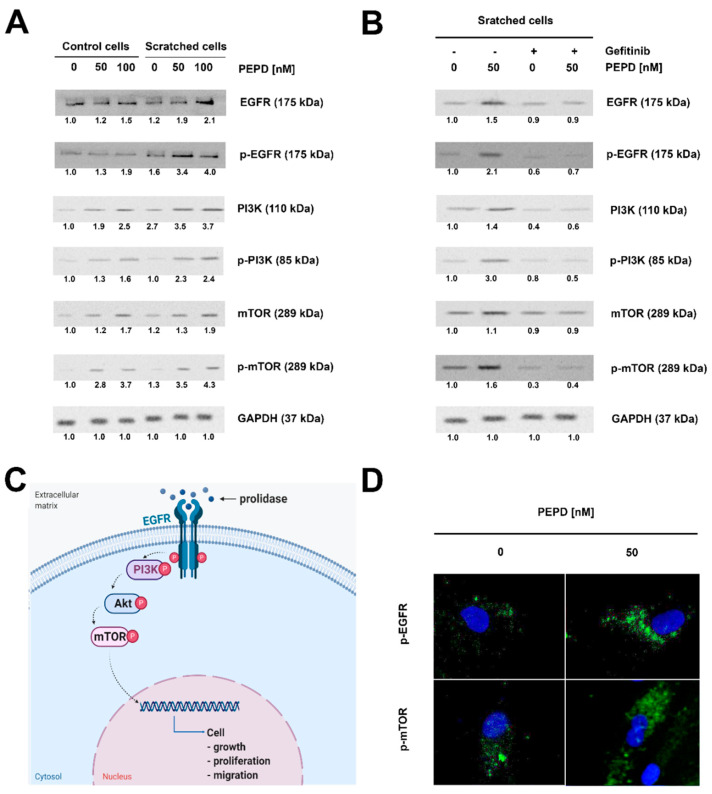
Extracellular PEPD-induced epidermal growth factor receptor (EGFR)-downstream signaling pathway. (**A**) Western blot for the proteins of EGFR-downstream signaling pathway in lysates of control and “scratched” PEPD-treated fibroblasts (PEPD, 1−100 nM) for 24 h or PEPD-treated fibroblasts (PEPD, 0 and 50 nM) with an inhibitor of EGFR (Gefitinib pretreated cells for 2 h, 0 and 45 µM) for 24 h. GAPDH was used as a loading control. (**B**) Representative blot images were shown (densitometry of protein stains is presented under protein bands as a ratio versus control; Supplementary Figure S1). GAPDH was used as a loading control. (**C**) Illustration of the PEPD-dependent EGFR-downstream signaling pathway. Created with BioRender.com. (**D**) Representative results of immunostaining of p-EGFR and p-mTOR in PEPD-stimulated fibroblasts (50 nM) for 24 h are presented; magnification 200×.

To test whether extracellular PEPD functions may undergo via EGFR, the expression of EGFR-dependent signaling proteins was assessed in fibroblasts after PEPD treatment. As presented in Figure 3A, in PEPD-treated cells (50, and 100 nM) the phosphorylated and total forms of EGFR, PI3K (p85), mTOR protein expressions were increased in a dose-dependent manner. The expression of phospho-EGFR and phospho-mTOR in a model of wounded cells stimulated by PEPD was confirmed by immunofluorescence analysis (Figure 3D).

**Figure 4:**
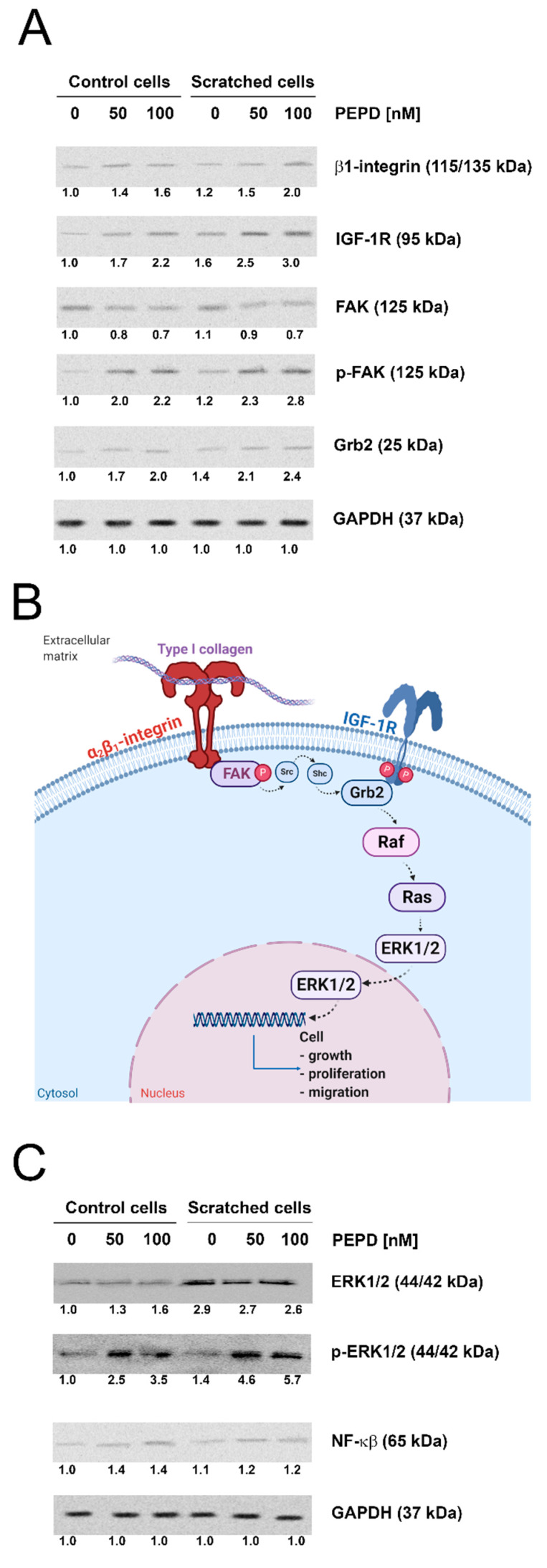
Extracellular PEPD induced expression of the β_1_-integrin receptor and IGF-1R signaling proteins in control and “scratched” fibroblast models. (**A**) The proteins of β_1_-integrin receptor and IGF-1R downstream signaling pathways, FAK, Grb2, and (**C**) NF-ĸβ and ERK1/2 were analyzed by Western blot in lysates of PEPD-treated fibroblasts (50, and 100 nM). Representative blot images were shown (densitometry of protein stains is presented under protein bands as a ratio versus control; Supplementary Figure S2). GAPDH was used as a loading control. (**B**) Illustration of the β_1_-integrin receptor-downstream signaling pathway. Created with BioRender.com.

Activation of EGFR signaling by PEPD is supported by the study showing that inhibition of EGFR function abolished the effects induced by PEPD. The cells were pretreated (2 h) with a well-known EGFR inhibitor, Gefitinib (final concentration: 45 µM). It was used to counteract PEPD-dependent stimulation of EGFR signaling. Gefinitib fully reduced PEPD-related phosphorylation of EGFR and diminished expression of total EGFR protein (Figure 4B). Figure 3C presents a potential PEPD-dependent activation of the EGFR-downstream signaling pathway in fibroblasts. 

### 2.4. The Expressions of the β_1_-integrin Receptor, IGF-1R, and Downstream Signaling Proteins Are Stimulated by an Extracellular PEPD in Control and “Scratched” Fibroblast Models

PEPD-treated fibroblasts showed elevated β_1_-integrin receptor and IGF-1R expressions. An increase in the expression of p-FAK and Grb2, downstream proteins to these receptors was also detected by Western blot (Figure 4A). The potential signaling pathway induced by studied receptors is mediated by Ras/Raf/ERK signaling as presented in Figure 4B. PEPD strongly stimulated p-ERK1/2 in cellular models of control and “scratched” fibroblasts. Interestingly, the expression of NF-ĸβ (an inhibitor of type I collagen gene expression) was elevated in PEPD-treated control cells, while “scratched” fibroblasts did not express the protein (Figure 4C). 

### 2.5. Collagen Biosynthesis Was Stimulated by Extracellular PEPD in Control and “Scratched” Fibroblast Models

**Figure 5:**
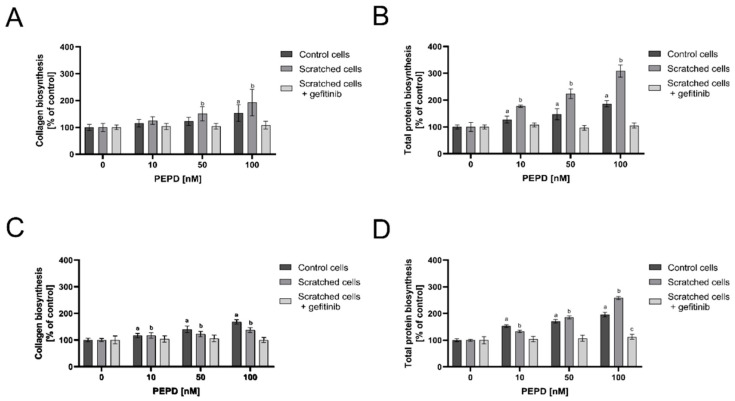
Extracellular PEPD activated collagen and total protein biosynthesis in control and “scratched” fibroblast models. Collagen biosynthesis (**A**,**C**) and total protein biosynthesis (**B**,**D**) in PEPD-treated fibroblasts (1–100 nM) in the presence and absence of EGFR inhibitor (Gefitinib, 0 and 45 µM pretreated cells for 2 h) after 24 and 48 h incubation, respectively. The values were presented as a percent of control cells (0 nM of PEPD). Mean values± SD of three experiments done in replicates is presented. The results are significant at a, b, c < 0.05, and are marked as a vs. control (0 nM of PEPD) of control cells, b vs. control (0 nM of PEPD) of scratched cells, c vs. control (0 nM of PEPD) of scratched cells incubated with gefitinib, respectively. PEPD—prolidase.

The effect of PEPD on collagen biosynthesis and total protein biosynthesis was measured by radiometric assay in control and “scratched” fibroblast models. The cells were treated with the selected concentrations of PEPD (10, 50, and 100 nM) for 24 h and 48 h. As shown in Figure 5A,C, extracellular PEPD stimulated collagen biosynthesis in both control and “wounded” cells in a manner dependent on used doses and time of incubation. A similar effect of PEPD was found concerning total protein synthesis. Figure 5B,D shows that total protein biosynthesis, which was used for normalization of the results of collagen biosynthesis, is stimulated by PEPD depending on used doses and time of incubation in both cell models. Inhibitor of EGFR (Gefitinib) abolished PEPD-dependent stimulation of collagen biosynthesis (A, C) and total protein biosynthesis (B, D) in studied cell model. The data suggest that the predominant portion of proteins synthesized due to PEPD stimulation is represented by collagen.

## 3. Discussion

The development of all organs requires fibroblasts to synthesize connective tissue constituents and maintain tissue architecture. The cells are of special interest in the repair phase of wound healing, since fibroblasts migrate into inflammatory sites, proliferate, and produce extracellular matrix components as glycosaminoglycans and collagen for scar formation. It is well established that migration of fibroblasts can be elicited by a variety of growth factors, e.g., EGF, IGF-1, PDGF [4].

The hypothesis that the activation of EGFR by PEPD [6] may play a crucial role in tissue regeneration led us to investigate the functional significance of fibroblast proliferation and migration in an experimental model of wound healing. In this report, we provide evidence for PEPD-induced EGFR signaling, cell proliferation, and migration in the experimental model of mechanically wounded fibroblasts in vitro. 

It seems that PEPD is a good candidate as an activator of EGFR-dependent regeneration processes. It did not affect cell viability or cell membrane integrity in control and “wounded” fibroblast models. However, we observed that PEPD induced fibroblast proliferation in a dose- and time-dependent fashion as detected by evaluation of DNA biosynthesis. Interestingly, DNA biosynthesis was augmented in “scratched” cells, compared to control. Moreover, we observed that PEPD remarkably accelerated the migration of cultured human fibroblasts. In this case, we also noticed that PEPD-induced migration was dose- and time-dependent.

The mechanism explaining this process was related to the EGFR signaling. It is well established that EGFR activation is followed by an up-regulation of 3 signaling pathways such as PI3K/Akt/mTOR, the Ras/Raf/ERK, and JAK/STAT [6,26,27]. Our study shows that extracellular PEPD activated PI3K/Akt/mTOR proteins. The evidence for the mechanism was proved in the experiment showing that preincubation of the cells with EGFR inhibitor, Gefitinib, abolished the stimulatory effect of PEPD on total and phosphorylated forms of EGFR downstream signaling proteins as PI3K, Akt, and mTOR. These findings confirmed that PEPD binds to EGFR and evokes growth-promoting activity in the fibroblast model of wound healing. These data are supported by studies of Lee et al. [32] showing that blockade of the PI3K/Akt/mTOR pathway diminished cell proliferation and migration. 

Growth factor signaling is often coupled to signaling induced by adhesion receptors. The example is IGF-1 and α2β1 integrin receptor that is activated by collagen type I [9]. The communication between both types of receptors is called cross-talk. The activation of the β_1_-integrin receptor and IGF-1 triggers the signaling pathway cascade by the proteins FAK and MAPK (ERK1 and ERK2) [33]. PEPD strongly induced expressions of both receptors (β_1_-integrin and IGF-1R) and their downstream proteins, as FAK and ERK1/2. This signaling cascade is known to stimulate the biosynthesis of ECM constituents, especially collagen [8,34,35]. We have shown that up-regulation of ERK contributed to down-regulation of NF-ĸβ expression, a well-known inhibitor of collagen biosynthesis [36]. Therefore, the increase in collagen biosynthesis in PEPD-treated fibroblasts is a result of collagen biosynthesis stimulation by IGF-1R and inhibition of NF-ĸβ expression, as an inhibitor of collagen gene expression. These data are supported by other researches [36,37,38]. It cannot be excluded that in the studied cells collagen biosynthesis is stimulated by EGFR signaling. The experiment with Gefitinib proved such a possibility. The stimulatory effect of EGF on collagen biosynthesis was confirmed by other authors [39,40,41]. 

The functional significance of our findings could be of importance not only in wound healing but also in prolidase deficiency (PD). It is a rare autosomal recessive disorder that is described by massive imidodipeptiduria, skin lesions as well as elevated dipeptides contained proline in plasma [5,42,43,44,45,46,47]. The most specific manifestation of PD concerns connective tissue metabolism. All cases of PD are characterized by skin lesions (e.g., diffuse telangiectasia, purpuric rash, crusting erythematous dermatitis, progressive ulcerative dermatitis, particularly on the lower legs). PD results from low or lack of PEPD activity due to mutations in the *PEPD* gene [48,49,50]. Studies so far on PD were focused on the intracellular role of PEPD since its extracellular function was described just a few years ago. Given our data, it cannot be excluded that the described manifestation of PD may derive from a deficiency of extracellular PEPD function since supplementation of PD patients with proline or proline-convertible amino acids was ineffective in the therapy of the disease [51]. New data on the role of PEPD as a regulator of p53 function, interferon-α/β receptor maturation, and activation of EGFR or HER2 create the prospect of discovering new functions of PEPD [30]. As our study evidenced promising effects of PEPD in cell proliferation, migration, and connective tissue rearrangement (mainly on collagen biosynthesis), further experiments are crucial to understanding its role in PD and other connective tissue disturbances. 

## 4. Materials and Methods

### 4.1. Fibroblasts Cell Cultures

Fibroblasts cells were cultured as we described previously [16]. Fibroblasts were subjected to treatment with porcine kidney prolidase (Sigma-Aldrich, Saint Louis, MO, USA) at a concentration of 1–100 nM. Moreover, cells were pretreated with an EGFR inhibitor, Gefitinib (Sigma-Aldrich, Saint Louis, MO, USA) at a final concentration of 45 µM for 2 h before supplementation with prolidase. 

### 4.2. Cell Viability

The cell viability of treated cells was measured using the MTT assay, as described previously [16]. Cells survival was calculated as a percentage of living cells when compared to control (0 nM of PEPD, 100% survival).

### 4.3. NRU Assay

Neutral red uptake (NRU) assay was performed according to the protocol by Borenfreund and Puerner [52] to elucidate the permeability of prolidase treated cells. At indicated time-points, the culture medium was removed, after washed cells, Neutral Red solution (final concentration: 50 µg/mL; Sigma-Aldrich, Saint Louis, MO, USA) was added. After 30 min, the cells were washed, dye from viable cells was released by extraction with a mixture of acetic acid, ethanol, and water (1:50:49, respectively; Sigma-Aldrich, Saint Louis, MO, USA). After shaking, the absorbance was measured at 540 nm in a microplate reader (Asys UVN 340 microplate reader, Biochrom, Cambridge, UK) using a blank as a reference. Cytotoxicity was calculated as a percentage of the control (0 nM of PEPD, 100% of intact membranes).

### 4.4. Cell Proliferation Assay

The effect of extracellular prolidase on proliferation capability and DNA biosynthesis in treated cells was assessed using the CyQUANT Cell Proliferation Assay Kit (Thermo Fisher Scientific, Waltham, MA, USA) according to the manufacturer’s guidelines. At the indicated times, the culture medium from treated cells was discarded, cells were washed with PBS, and plates were frozen. Then, samples were thawed at room temperature and lysed using CyQUANT dye mix, and total cellular nucleic acid was measured by fluorometer at 480/520 nm wavelengths (VICTOR™ X4 Multilabel Plate Reader, PerkinElmer, MA, USA). The results were calculated as a percent of the control value. 

### 4.5. Total Protein and Collagen Biosynthesis

The cells were cultured in 6-well plates at 1 × 10^6^ cells/well with 2 mL of growth medium. After 48 h, the cells were incubated with 5[^3^H]-proline (5 μCi/mL; Hartmann Analytic, Braunschweig, Germany) and prolidase (1–100 nM) for 24 or 48 h. Total protein biosynthesis and collagen biosynthesis were measured by the incorporation of radioactive proline into proteins. Then, collagen was subjected to digestion by purified *Clostridium histolyticum* collagenase (Sigma-Aldrich, Saint Louis, MO, USA), according to the method of Peterkofsky et al. [53]. After isolation of proteins, the incorporation of tracer was measured in total proteins and collagenase-digestible proteins. The results were shown as combined values for cell plus medium fractions. 

### 4.6. Western Blot

The cells, after incubation with PEPD (50–100 nM) for 40 min and 24 h (respectively), were washed and then incubated on ice for 15 min with a mixture of lysis buffer (Cell Signaling Technology, Danvers, MA, USA) and a mix of protease inhibitors (Protease Inhibitors Mix G, SERVA, Heidelberg, Germany). Lysates were sonicated, centrifuged, and then the supernatant was stored at −80 °C until Western blot assay. The total concentration of protein was measured by the method of Lowry et al. [54]. The procedure of Western blot analysis was described previously [55]. Equal amounts (30 µg/lane) of protein were diluted in lysis buffer. Cell lysates were subjected to SDS-PAGE electrophoresis. After semi-dry transfer, membranes were blocked with non-fat dry milk in TBS-T. The membranes were incubated with primary antibodies (all from CST and in 1:1000 dilution; Cell Signaling, Danvers, MA, USA) overnight, including anti-EGF Receptor, anti-phospho-EGF Receptor, anti-PI3 Kinase p85, anti-phospho-PI3 Kinase p85, anti-mTOR, anti-phospho-mTOR, anti-Integrin β1 Receptor, anti-IGF-1 Receptor β, anti-FAK, anti-phospho-FAK, anti-Grb2 (in 1:1000 dilution, Becton, Dickson and Company, Franklin Lakes, NJ, USA), anti-NF-κβ p65, anti-p44/42 MAPK (ERK1/2), anti-phospho-p44/42 MAPK (ERK1/2), anti-GAPDH. Then membranes were washed and incubated with anti-mouse or anti-rabbit HRP-linked secondary antibodies at concentration 1:7500 (Sigma-Aldrich, Saint Louis, MO, USA). Then, the membranes were incubated with Amersham ECL Western Blotting Detection Reagent, (GE Healthcare Life Sciences, Helsinki, Finland) followed by an image capturing performed using the BioSpectrum Imaging System UVP (Ultra-Violet Products Ltd., Cambridge, UK). The band intensity was measured by ImageJ software (https://imagej.nih.gov/ij/). Western blot analysis was performed at least in triplicates. 

### 4.7. In Vitro Wound-Healing Assay

An analysis of cell migration was conducted using an in vitro wound-healing assay. Fibroblasts were cultured in six-well plates to confluency, then they were scratched with a sterile 200 μL pipette tip. Before PEPD treatment, the cell monolayer was rinsed three times with PBS. The cells were treated with PEPD concentrations in the range 10–50 nM for 24 and 48 h. Image capture of cells (at least in triplicates) was made using an inverted optical microscope (Nikon, Tokyo, Japan) every 24 h with a 40× magnification to monitor the wound closure. The wound closure was counted by ImageJ software (https://imagej.nih.gov/ij/) and calculated according to the following formula [56]. 

### 4.8. Immunocytochemistry

Immunocytochemistry was conducted according to BDB Bioimaging protocol, as described previously [55]. Cells grown on a 96-wells plate were fixed with paraformaldehyde, then permeabilized with Triton and blocked with 3% foetal bovine serum. Cells were incubated with primary antibodies (anti-phospho-EGFR and anti-phospho-mTOR, dilution 1:1000), then with FITC-linked secondary antibody and Hoechst. A confocal laser scanning microscope (BD Pathway 855 Bioimager; Becton, Dickson and Company, Franklin Lakes, NJ, USA) with AttoVision software was used for image capture.

### 4.9. Statistical Analysis

All experiments were run at least in triplicates and the experiments were repeated twice. Data represent a mean ± standard deviation (SD). For statistical analysis, we used one-way analysis of variance (ANOVA) with Dunnett’s correction and *t*-test using GraphPad Prism 5.01 (GraphPad Software, San Diego, CA, USA). 

## 5. Conclusions

**Figure 6:**
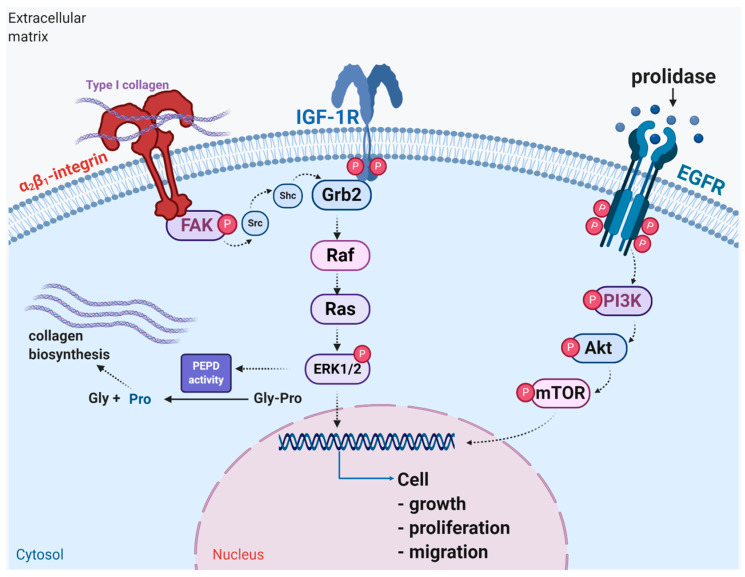
Schematic representation of the functional significance of activation of EGFR, IGF-1R, and β_1_-integrin receptor downstream signaling in wound healing. Under experimental conditions of mechanically “wounded” fibroblasts, PEPD activates EGFR-dependent downstream PI3K/Akt/mTOR signaling, while IGF-1R and β_1_-integrin receptor cooperate to activate MAPK (ERK1/2) pathway resulting in increased cell proliferation, migration and collagen biosynthesis. Created with BioRender.com.

In this report, we demonstrated results presenting that extracellular PEPD binding to EGFR accelerates wound healing in cultured fibroblasts. The potential mechanism is outlined in Figure 6. It suggests that PEPD may be considered as a therapeutic agent for skin wound healing.

## Data Availability

The datasets used and/or analyzed during the current study are available from the corresponding author on reasonable request.

## Supplementary Materials

Supplementary materials can be found at https://www.mdpi.com/1422-0067/22/2/942/s1.

## Abbreviations

Akt	protein kinase B
ECM	extracellular matrix
EGF	epidermal growth factor
EGFR	epidermal growth factor receptor
ERBB	family of proteins contains four receptor tyrosine kinases
ERK1/2	extracellular signal-regulated kinase 1/2
FAK	focal adhesion kinase pp125^FAK^
GAPDH	glyceraldehyde 3-phosphate dehydrogenase
Grb2	growth factor receptor-bound protein 2
HER 2	epidermal growth factor receptor 2
IGF-1	insulin-like growth factor 1
IGF-1R	insulin-like growth factor 1 receptor
JAK	Janus kinase
MAPK	mitogen-activated protein kinase
MEK	mitogen-activated protein kinase kinase
mTOR	mammalian target of rapamycin
NF-κβ	nuclear factor kappa beta
p53	tumor protein p53
PD	prolidase deficiency
PDGF	platelet-derived growth factor
PEPD	prolidase
PI3K	phosphoinositide 3 kinase
Pro	proline
Shc	SHC adaptor protein 1
Sos1	son of sevenless 1
STAT	signal transducer and activator of transcription
Src	proto-oncogene Src
