# Supplementary material for: Extracellular Prolidase (PEPD) Induces Anabolic Processes through EGFR, β1-integrin, and IGF-1R Signaling Pathways in an Experimental Model of Wounded Fibroblasts
Source: Int J Mol Sci. 2021 Jan 19;22(2):942. doi: 10.3390/ijms22020942 (PMC7833428; doi:10.3390/ijms22020942)

File name: Additional file 1

Title of data: Supplementary data analysis presented on Figure 3 and Figure 4.

Description of data: The blots and images are described in the result section.SFig. 1. - Supplementary data analysis presented on Figure 3.Representative blots from Western blotting analysis presented in Figure 3A.

1.1.1. EGFR expression and its intensity

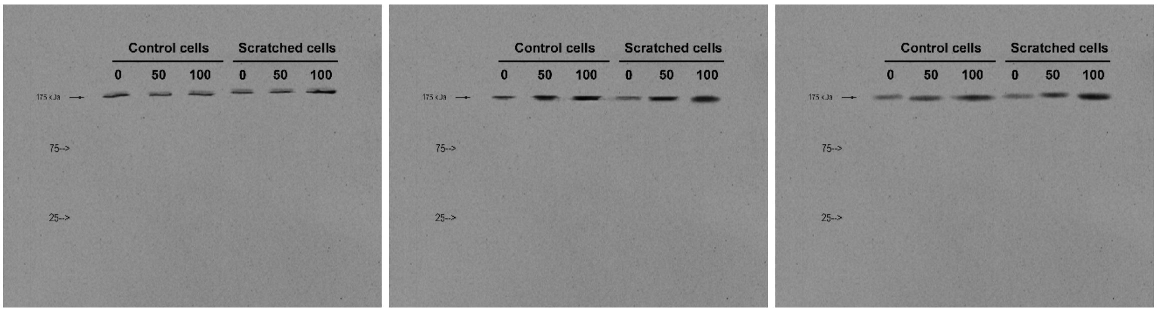

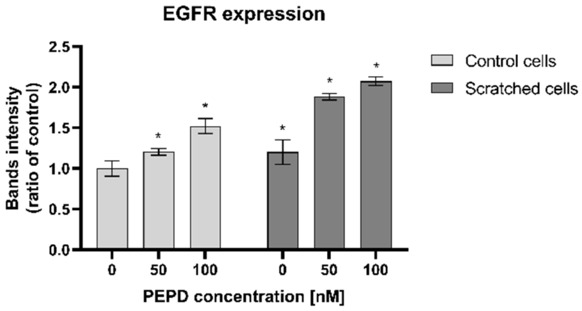

1.1.2. Phospho-EGFR expression and its intensity

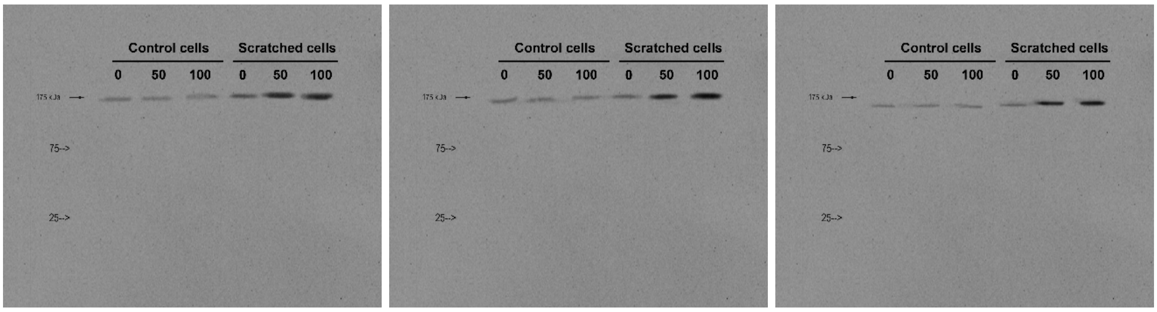

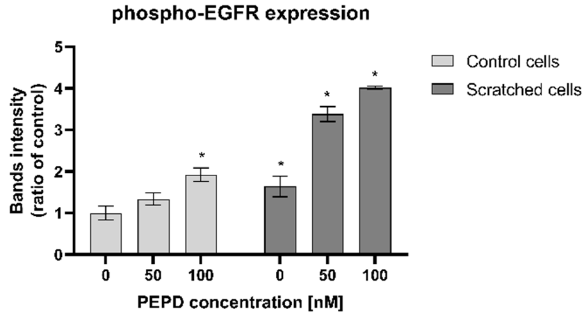

1.1.3. Ratio of phosphor-EGFR/EGFR expression

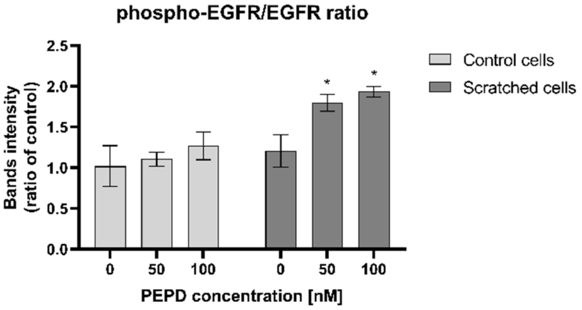

1.1.4. PI3K expression and its intensity

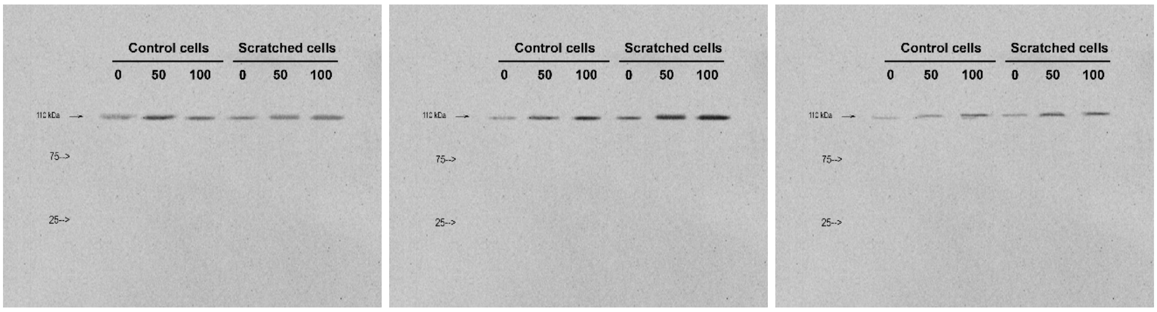

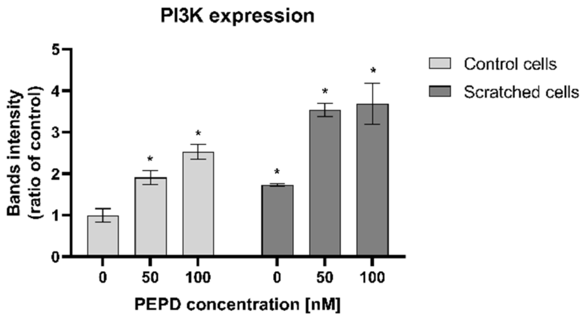

1.1.5. Phospho-PI3K expression and its intensity

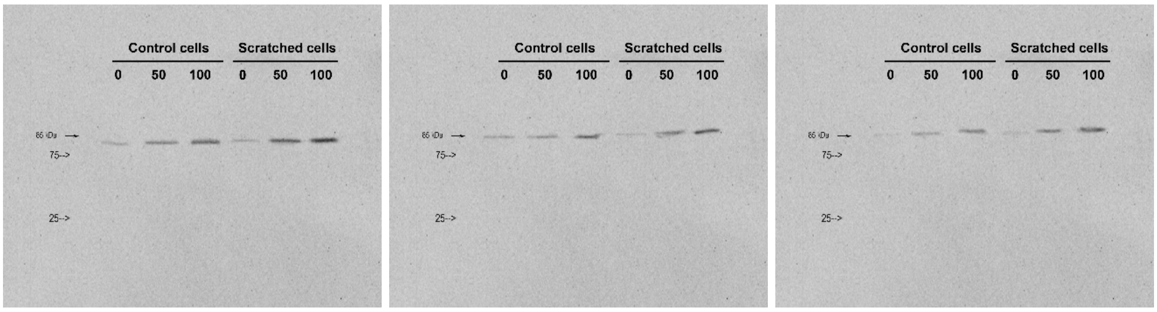

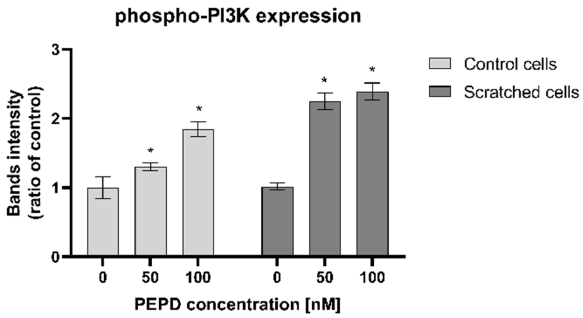

1.1.6. Ratio of phospho-PI3K/PI3K expression

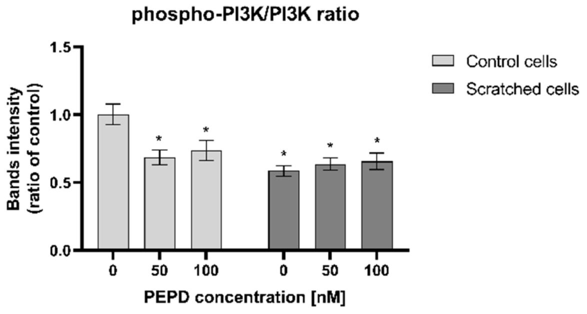

1.1.7. mTOR expression and its intensity

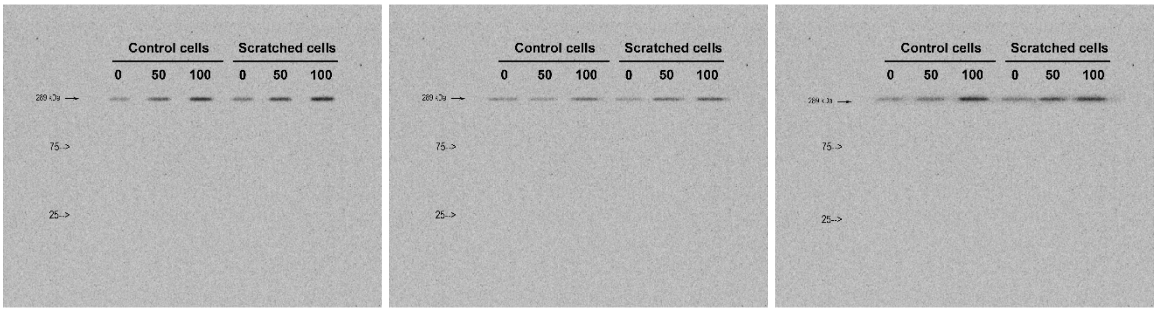

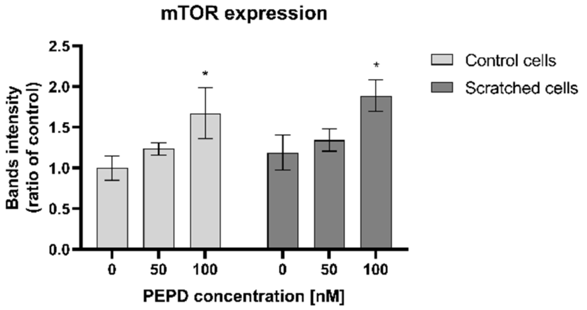

1.1.8. Phospho-mTOR expression and its intensity

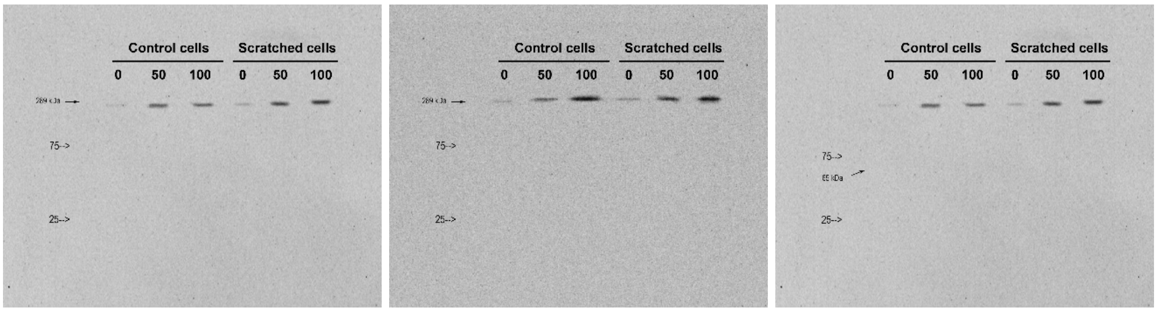

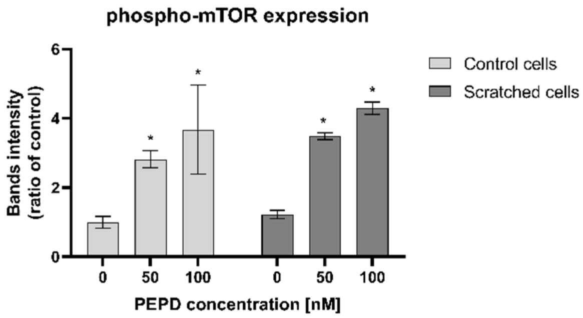

1.1.9. Ratio of phosphor-mTOR/mTOR expression

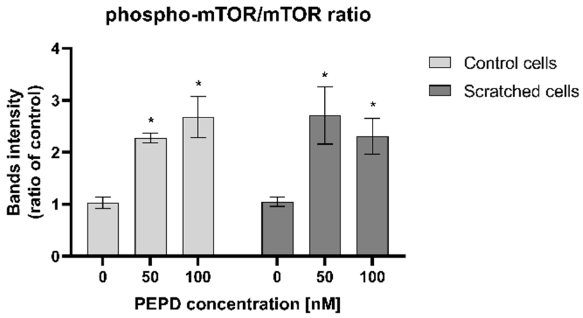

1.2.Representative blots from Western blotting analysis presented in Figure 3B

1.2.1. EGFR expression and its intensity

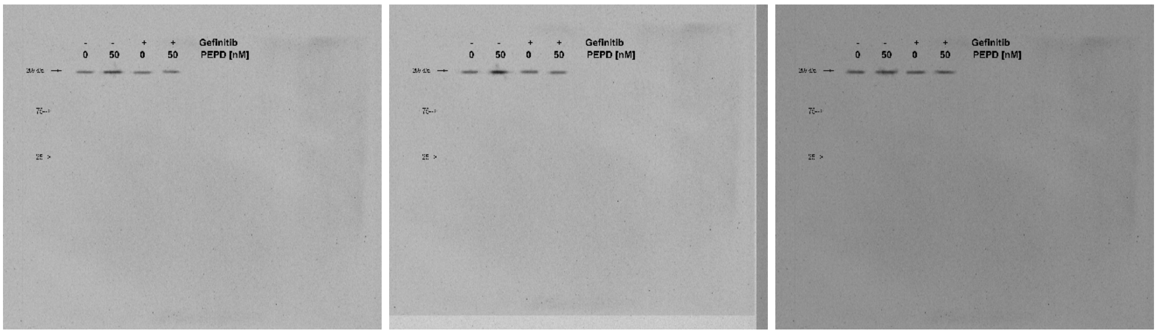

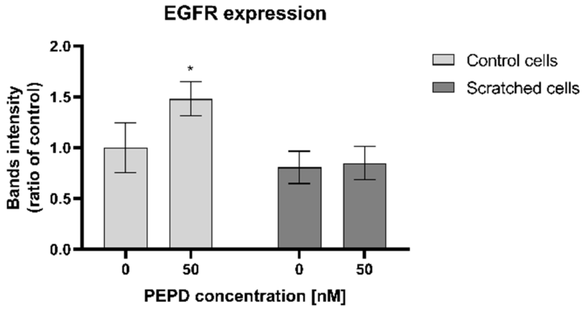

1.2.2. Phospho-EGFR expression and its intensity

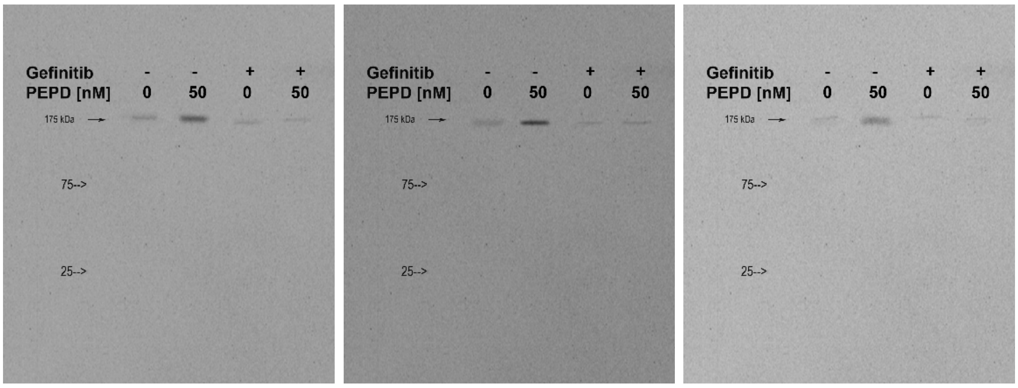

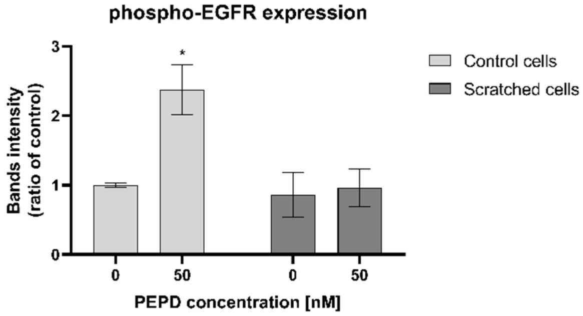

1.2.3. Ratio of phosphor-EGFR/EGFR expression

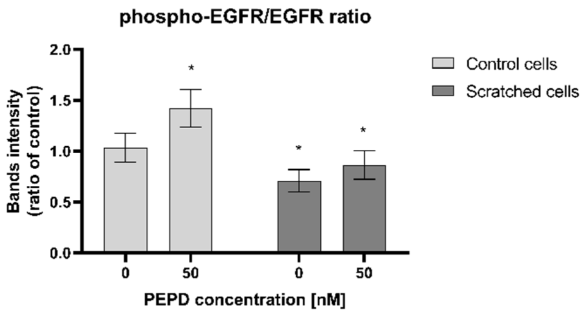

1.2.4. PI3K expression and its intensity

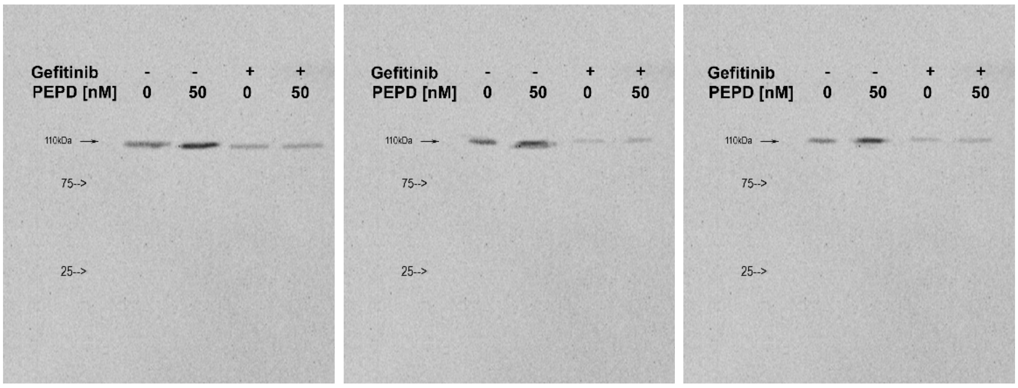

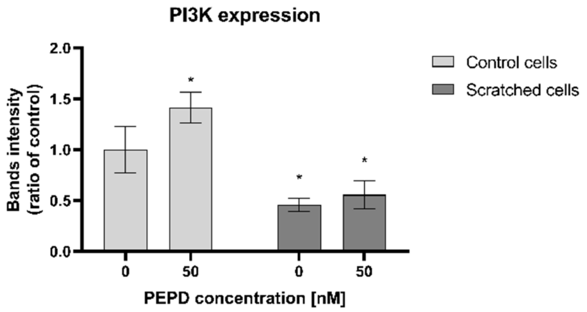

1.2.5. Phospho-PI3K expression and its intensity

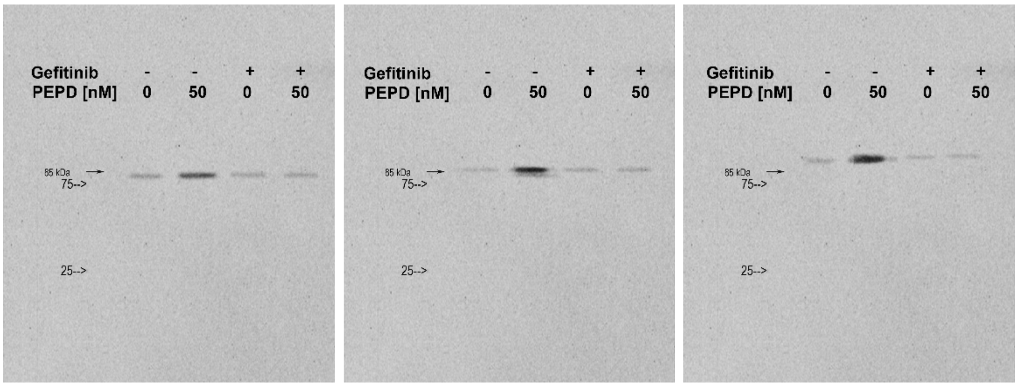

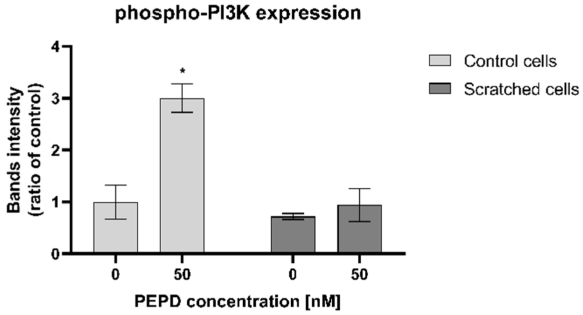

1.2.6. Ratio of phospho-PI3K/PI3K expression

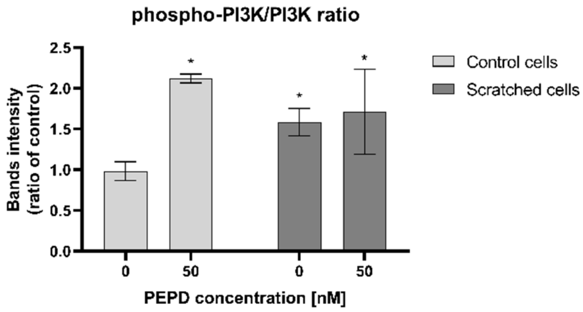

1.2.7. mTOR expression and its intensity

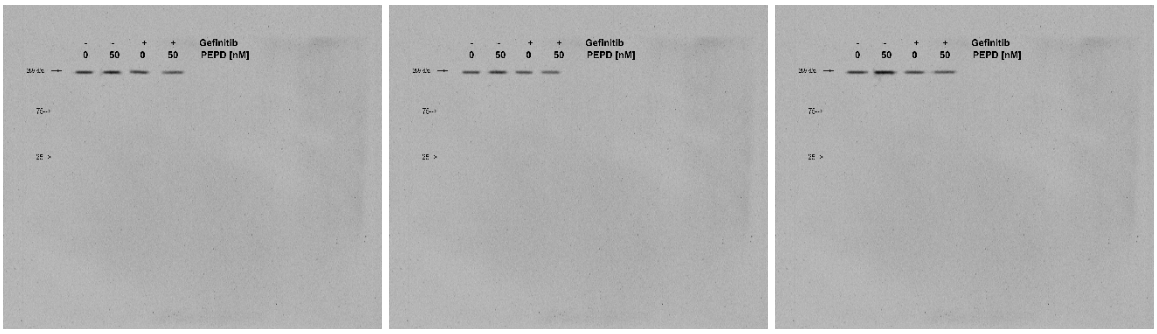

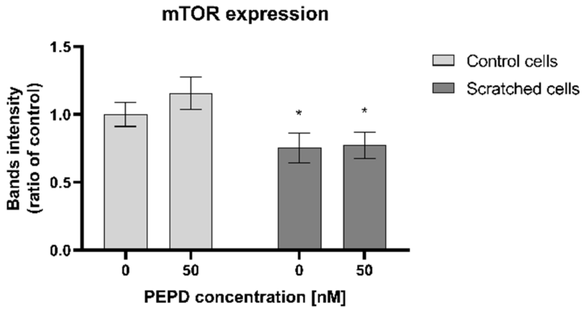

1.2.8. Phospho-mTOR expression and its intensity

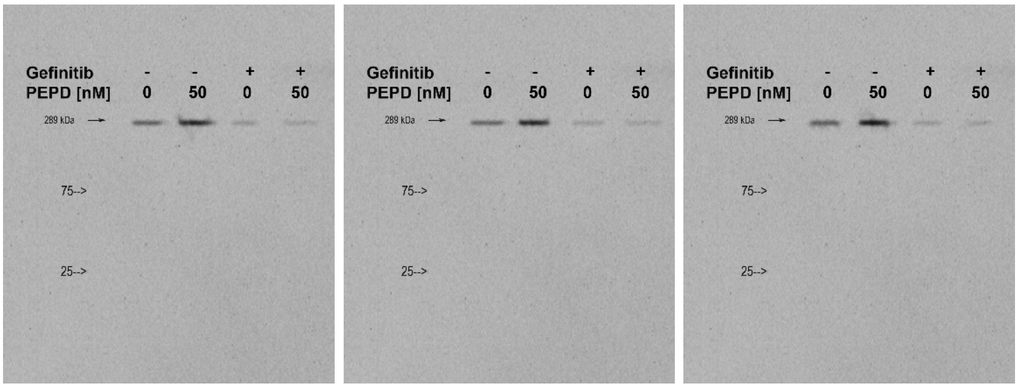

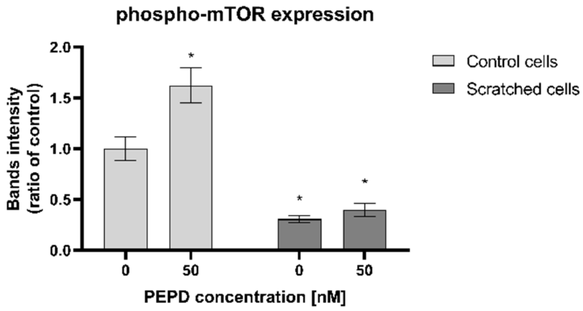

1.2.9. Ratio of phosphor-mTOR/mTOR expression

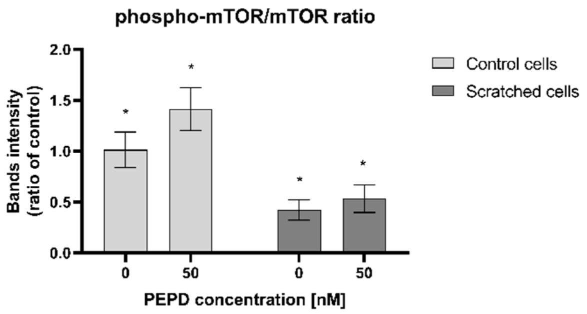

2. SFig.2. - Supplementary data analysis presented on Figure 4

2.1.Representative blots from Western blotting analysis presented in Figure 4A

2.1.1. B1-integrin expression and its intensity

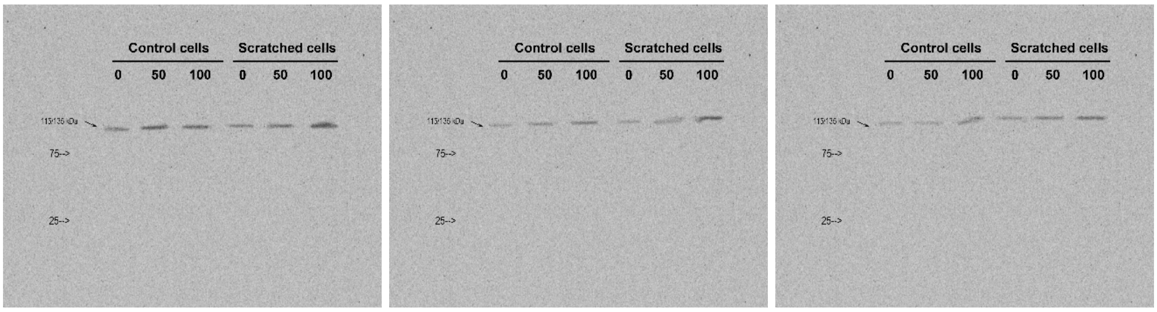

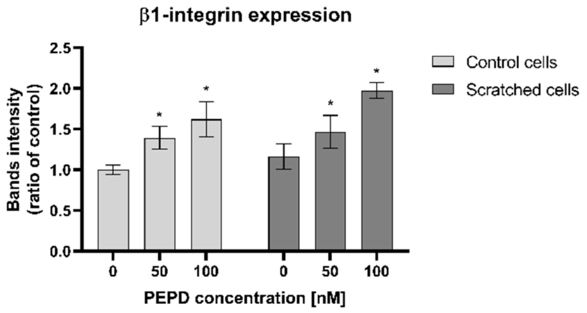

2.1.2. IGF-1R expression and its intensity

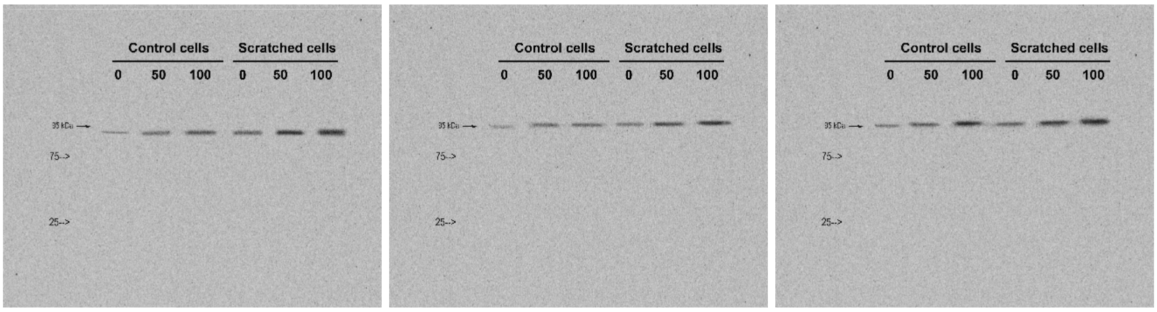

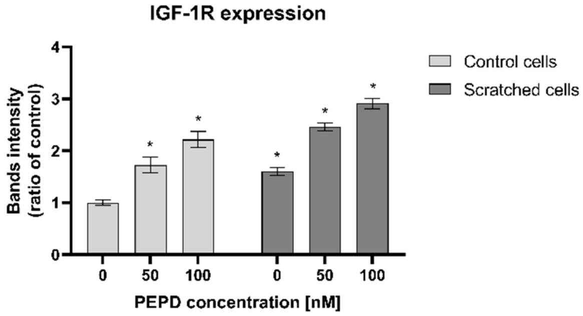

2.1.3. FAK expression and its intensity

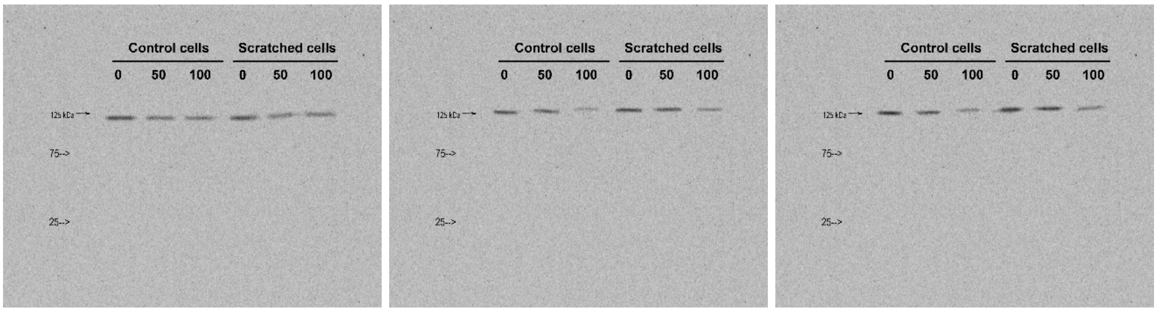

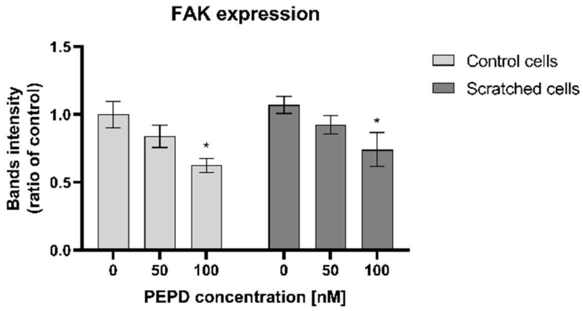

2.1.4. Phospho-FAK expression and its intensity

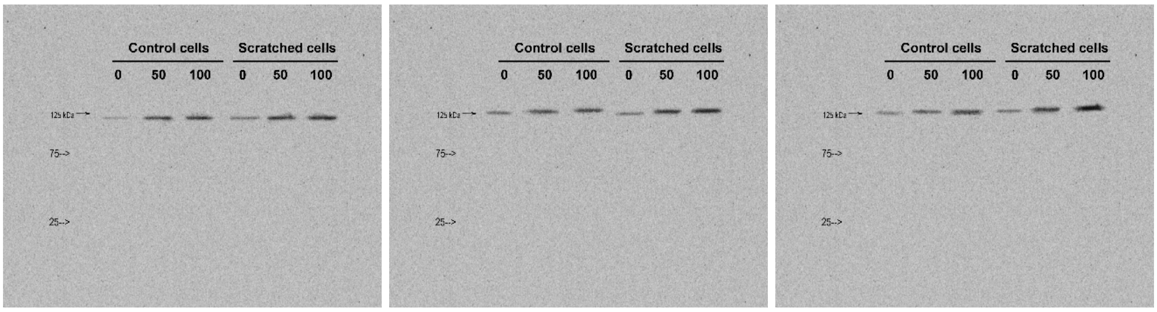

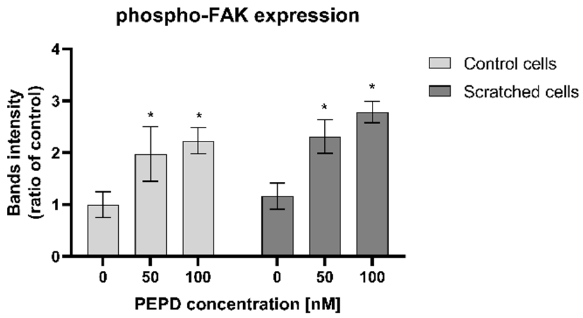

2.1.5. Ratio of phospho-FAK/FAK expression

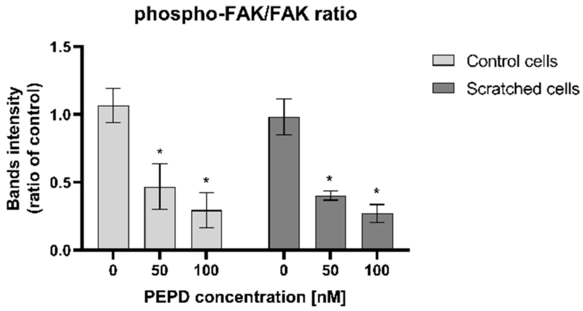

2.1.6. Grb2 expression and its intensity

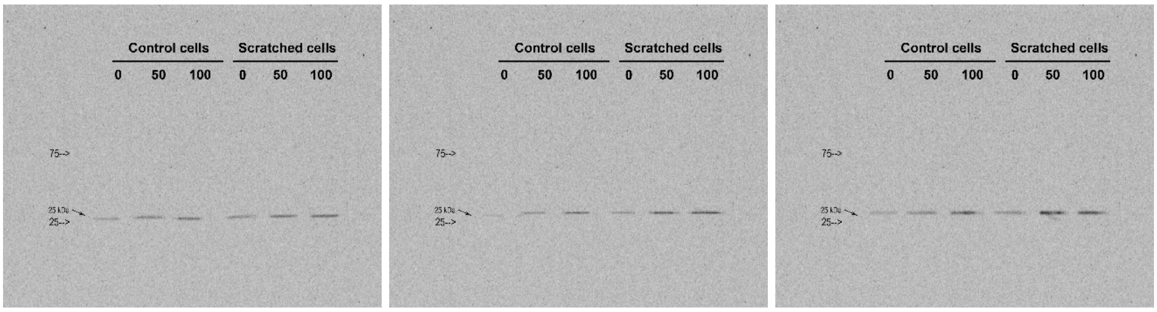

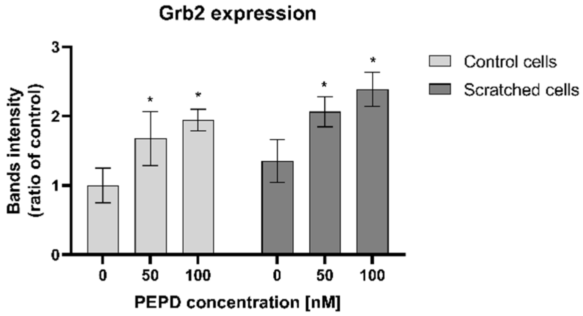

2.2. Representative blots from Western blotting analysis presented in Figure 4C

2.2.1. ERK expression and its intensity

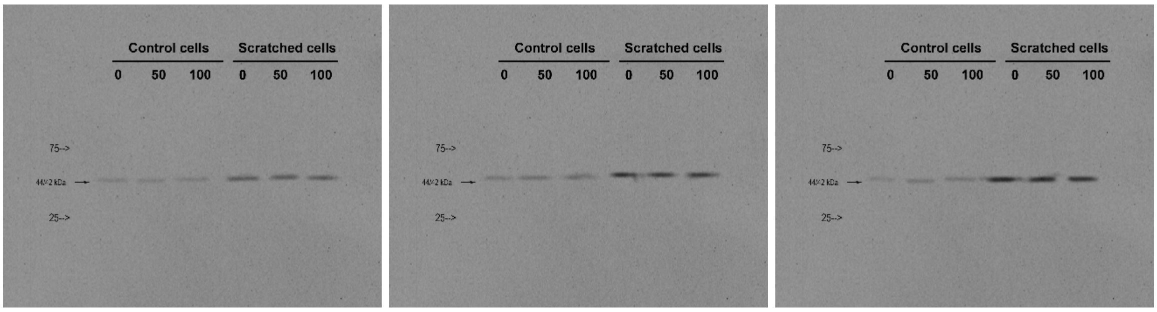

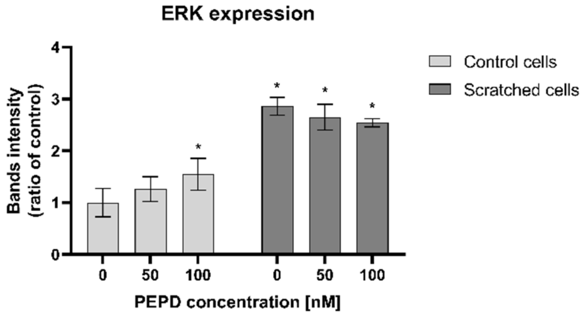

2.2.2. Phospho-ERK expression and its intensity

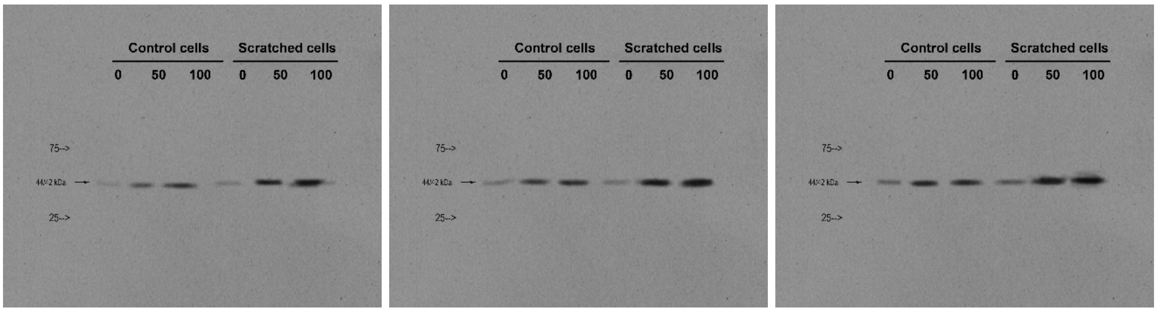

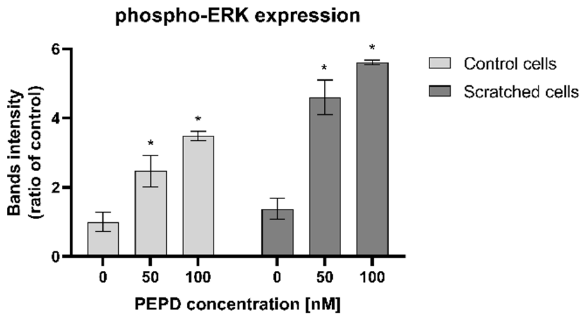

2.2.3. Ratio of phosphor-ERK/ERK expression

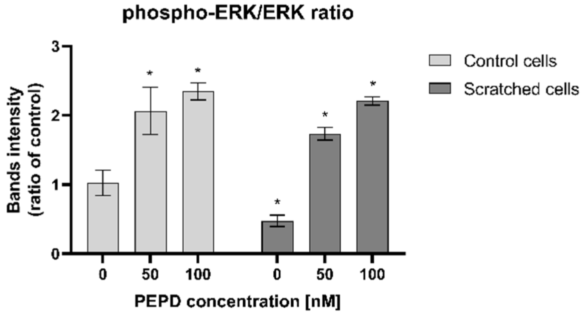

2.2.4. NF-κB expression and its intensity

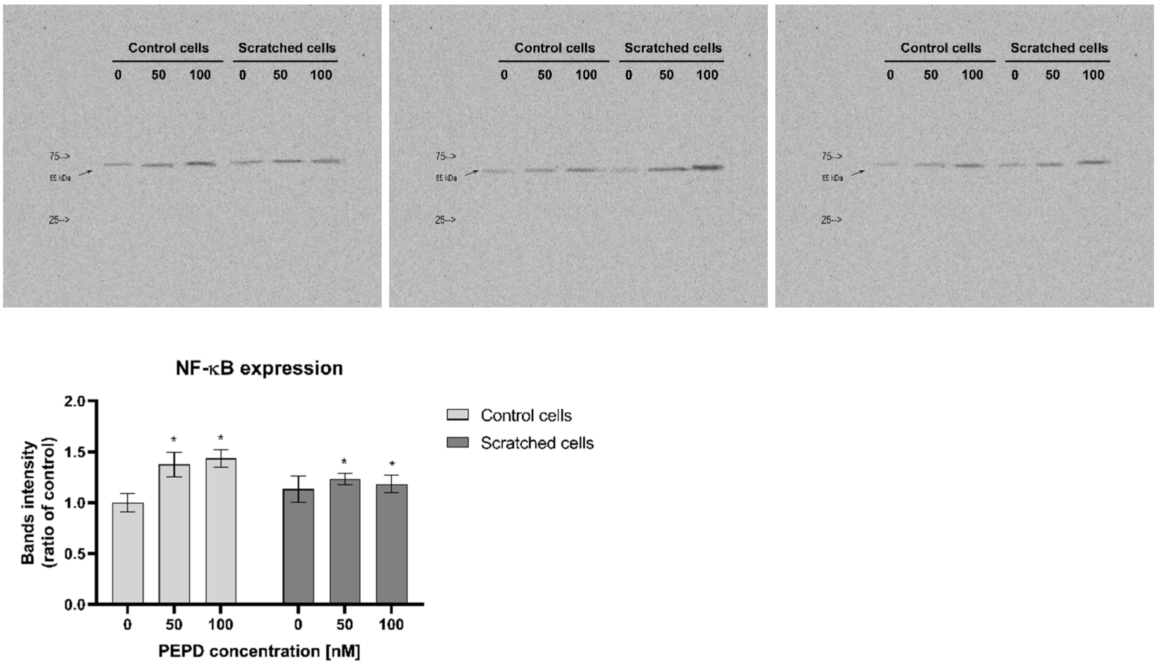

2.3.Representative blots of GAPDH from Western blotting analysis

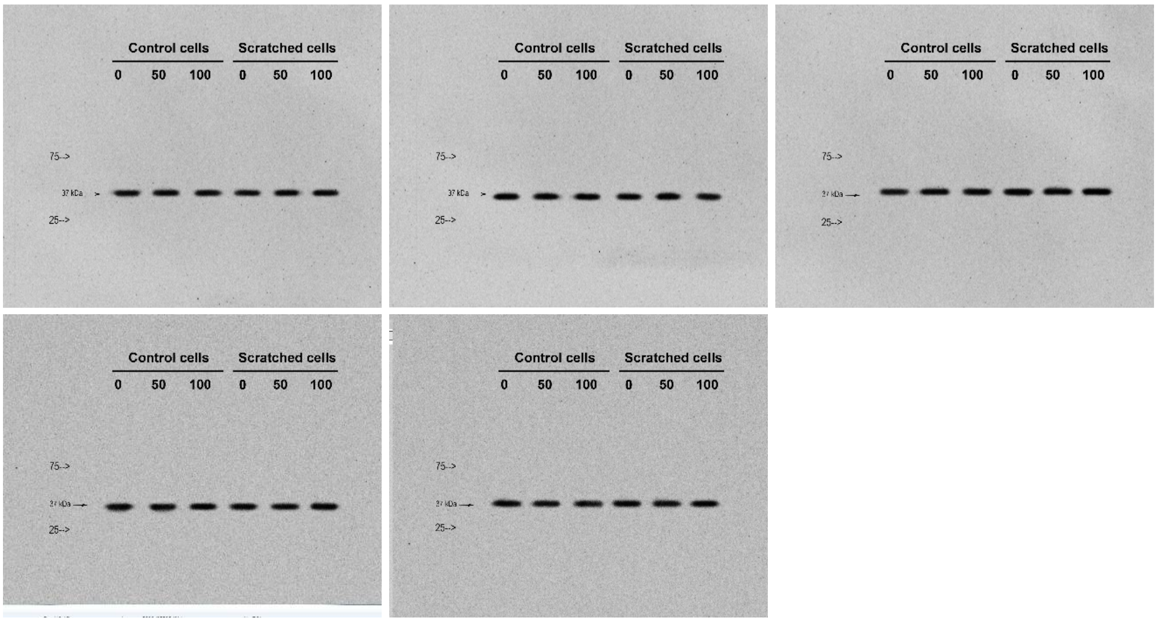

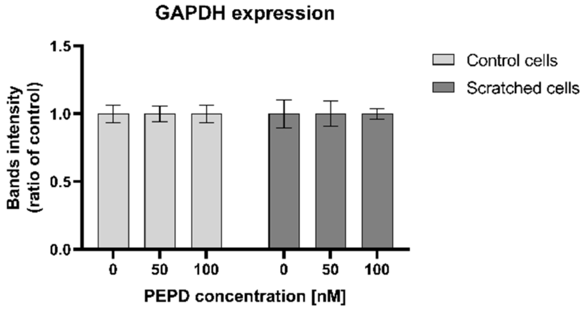

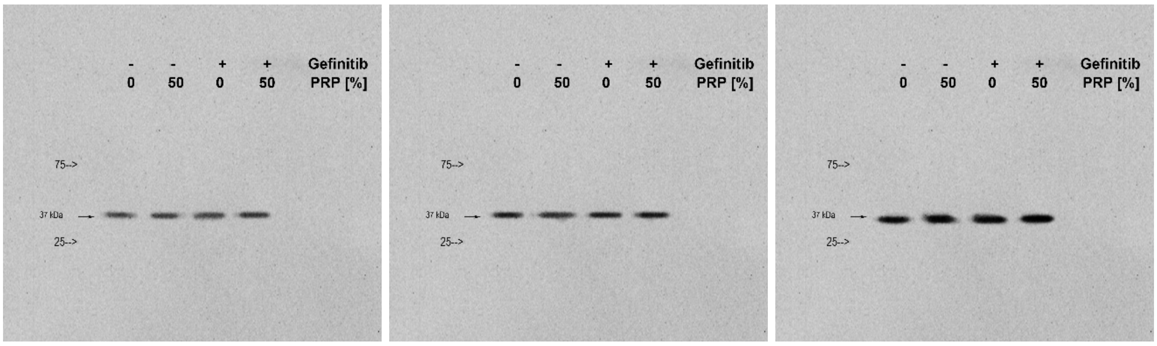

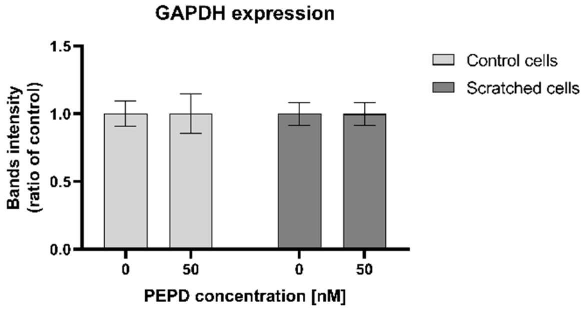

Supplement: Supplementary file 1 [file ijms-22-00942-s001.pdf]
